# Effects of psychedelic use on authoritarian attitudes revisited

**DOI:** 10.1177/02698811261443677

**Published:** 2026-05-02

**Authors:** Otto Simonsson, Taylor Lyons, Joseph Marks, Hannes Kettner, Leor Roseman, Eline Haijen, Mendel Kaelen, Robin Carhart-Harris

**Affiliations:** 1 Center for Social Sustainability, Department of Neurobiology, Care Sciences and Society, Karolinska Institutet, Stockholm, Sweden; 2 Centre for Psychedelic Research, Imperial College London, UK; 3 Department of Experimental Psychology, University College London, UK; 4 Department of Psychology, University of Exeter, UK; 5 Department of Neuropsychology and Psychopharmacology, Faculty of Psychology and Neuroscience, Maastricht University, the Netherlands; 6 Department of Neurology, University of California, San Francisco, CA, USA

**Keywords:** psychedelics, authoritarian, political, psilocybin, LSD

## Abstract

**Background:**

Previous research suggests that psychedelics may, under certain conditions, decrease authoritarian attitudes, but larger and more rigorously designed studies are needed to confirm these findings.

**Aims:**

We aimed to examine the effects of psychedelic use on authoritarian attitudes.

**Methods:**

Using data from three separate studies with different designs and populations, we investigated the relationship between psychedelic use and authoritarian attitudes. Study 1 was a naturalistic observational study with participants who planned to use psychedelics at their own initiative, Study 2 was a single-arm study with healthy volunteers who received psilocybin, and Study 3 was a randomized, controlled trial with patients diagnosed with depression who received psilocybin or escitalopram.

**Results:**

Across the three studies, results showed no significant changes in authoritarian attitudes after psychedelic use.

**Conclusions:**

Contrary to previous research, the latest evidence is not compelling that psychedelic use influences authoritarian attitudes in a reliable direction. Future research should recruit larger and more diverse samples, collect additional context-related data, and also investigate political outcomes other than authoritarian attitudes.

## Introduction

Psychedelics are a group of psychoactive drugs that can reliably induce changes in perception and cognition at certain doses (Nichols, 2016). While the definition of psychedelics typically includes 5HT2A receptor agonists such as psilocybin and lysergic acid diethylamide (LSD; Nichols et al., 2023), other compounds with quite different pharmacological profiles (e.g., Salvia divinorum, ibogaine, 5-MeO-DMT) are sometimes classified as atypical psychedelics (Dourron et al., 2023; Siegel et al., 2021). These psychedelic substances may all share an ability to increase psychological and neural plasticity (Carhart-Harris et al., 2015; Nardou et al., 2023), making the mind and brain more sensitive and open to both change and environmental influences (Brouwer and Carhart-Harris, 2021). The type of changes, however, that may occur following psychedelic use, as well as the direction of those changes, appear to be at least partially dependent on context (Carhart-Harris et al., 2018). It suggests that the effects of psychedelics can vary considerably depending on contextual factors.

The evidence to date suggests that psychedelics, under certain conditions, may provide clinical benefits across a range of psychiatric populations (Yao et al., 2024), but recent research indicates that these substances can potentially also influence non-clinical outcomes such as personality and political attitudes. For example, in a cross-sectional study of participants who had at least once tried psychedelics, cocaine or alcohol, both lifetime psychedelic use and degree of ego dissolution during the most intensive psychedelic experience were negatively correlated with authoritarian attitudes (Nour et al., 2017) – a construct that broadly captures endorsement of authoritary and social conformity. While the cross-sectional research design precluded determination of temporal order, another study with a longitudinal research design found a decrease in authoritarian attitudes following two dosing sessions of psilocybin (10 mg, 25 mg), paired with psychological support, among patients with treatment-resistant depression (Lyons and Carhart-Harris, 2018). Such results are noteworthy and yet should be considered in light of the study’s limitations, including its small sample size and its open-label, non-randomized design. The effects of psychedelic use on authoritarian attitudes should therefore be revisited and further investigated, ideally with bigger samples and more rigorous research designs.

In three exploratory studies with different designs and populations, we investigated the relationship between psychedelic use and authoritarian attitudes. In Study 1, using a prospective online study, we investigated associations between naturalistic psychedelic use and changes in authoritarian attitudes among participants who planned to use psychedelics at their own initiative. In Study 2, using a controlled, single-blind, within-subjects design, we investigated the potential effects of psilocybin, paired with psychological support, on authoritarian attitudes among healthy volunteers. In Study 3, using a double-blind randomized, controlled design, we investigated the effects of psilocybin, paired with psychological support, on authoritarian attitudes among patients with major depressive disorder.

## Methods and design

### Population and procedure

In Study 1, adult individuals who intended to use certain types of psychedelics (i.e., LSD or 1P-LSD, psilocybin, ayahuasca, DMT or 5-MeO-DMT, Salvia divinorum, mescaline, or ibogaine) in the near future could participate in the study. Recruitment occurred through online advertisements (e.g., posted and shared on Facebook, online drug forums, and email newsletters). The eligible individuals who wanted to participate in the study provided informed consent digitally and entered the expected date of their planned psychedelic experience, which ensured they received email links to the questionnaires at the appropriate time points. The data were collected as part of a larger study (Haijen et al., 2018). The study was approved by the Imperial College Research Ethics Committee and the Imperial College London Joint Research Compliance Office (see Haijen et al., 2018, for more information about the study).

In Study 2, adult individuals who did not have physical or mental health problems and who had not had any prior experience with psychedelics could participate in the study, as long as they did not meet any of the exclusion criteria (e.g., medically significant condition, current or previously diagnosed psychiatric disorder, immediate family members with a current or previously diagnosed psychotic disorder). Recruitment occurred through online advertisement on the homepage of the Centre for Psychedelic Research at Imperial College London. The eligible individuals who wanted to participate provided written informed consent and received two oral doses of psilocybin, 4 weeks apart. The first dose was 1 mg psilocybin, considered to be a subthreshold dose that is unable to occasion a psychedelic experience. The second dose took place 4 weeks later and was a fully active dose of 25 mg psilocybin, considered to be a high dose and capable of inducing a psychedelic experience. The participants were instructed to refrain from recreational drug use for the duration of the study and alcohol for at least 24 hours before each study visit. To uphold blinding and control for expectancy effects, participants were informed that they would receive psilocybin in both sessions with a variable dose up to 25 mg. No further information regarding dosage was provided. The data were collected as part of a larger study (Lyons et al., 2024). The study was approved by the London-Surrey Research Ethics Committee, the Imperial College London Joint Research Compliance Office, and other relevant authorities (see Lyons et al., 2024, for more information about the study).

In Study 3, adult individuals diagnosed with moderate-to- severe major depressive disorder could participate in the study, as long as they did not meet any of the exclusion criteria (e.g., medically significant condition, immediate family members with a current or previously diagnosed psychotic disorder). Recruitment occurred through trial networks, social media, and additional outreach channels. The eligible individuals who wanted to participate provided written informed consent and were informed that they would receive psilocybin, but the dose was not disclosed to standardize expectations. The participants were randomized (1:1 ratio) to an escitalopram condition or a psilocybin condition. The participants in the escitalopram condition received 1 mg of psilocybin on two occasions, 3 weeks apart, plus psychological support and 6 weeks of daily capsules containing 10 mg of escitalopram, increasing to ×2 capsules for the final 3 weeks (i.e., 20 mg escitalopram daily). The participants in the psilocybin condition received 25 mg of psilocybin on two occasions, 3 weeks apart, plus psychological support and 6 weeks of daily capsules containing inert placebo (i.e., microcrystalline cellulose). The data were collected as part of a larger study (Carhart-Harris et al., 2021). The study was approved by the Brent Research Ethics Committee, the Imperial College London Joint Research Compliance Office, and other relevant authorities (see Carhart-Harris et al., 2021, for more information about the study).

### Measure

The outcome measure across all three studies was authoritarian attitudes (Cronbach’s alpha between 0.62 and 0.70 at baseline in all three studies), which was measured using five survey items derived from a larger questionnaire (Evans et al., 1996): “Young people today don’t have enough respect for traditional values,” “People who break the law should be given stiffer sentences,” “Schools should teach children to obey authority,” “The law should always be obeyed, even if a particular law is wrong,” and “Organizing public meetings to protest against the government should not be allowed.” The 5-item questionnaire has been psychometrically evaluated and has been used in previous studies on psychedelic use and authoritarian attitudes (Lyons and Carhart-Harris, 2018; Nour et al., 2017). The responses were rated on a 1- (strongly disagree) to 5- (strongly agree) Likert scale. The total score was computed by averaging the scores of all survey items.

Because the three studies included slightly different assessment schedules, we focused on the time points that were roughly comparable across studies. In Study 1, authoritarian attitudes were measured at baseline (T1), 2-weeks post-psychedelic experience (T2), and 4-weeks post-psychedelic experience (T3). In Study 2, authoritarian attitudes were measured at baseline (T1), 2-weeks post-placebo experience (T2; 1 mg psilocybin), 4-weeks post-placebo experience (T3; 1 mg psilocybin), 2-weeks post-psilocybin experience (T4; 25 mg psilocybin), and 4-weeks post-psilocybin experience (T5; 25 mg psilocybin). In Study 3, authoritarian attitudes were assessed at baseline (T1) and 6-weeks post-baseline (T2).

### Statistical analysis

In Study 1, data were analyzed using a linear mixed-effects model (LME) with Bonferroni-corrected paired *t*-tests to test for differences in authoritarian attitudes at the 2-week (T2) and 4-week (T3) time points, relative to pre-experience baseline (T1) scores. In Study 2, data were analyzed using a LME with Bonferroni-corrected *post hoc* comparisons to test for differences in authoritarian attitudes between the time points relative to baseline scores. In Study 3, paired t-tests were performed to test for differences in authoritarian attitudes at the 6-week (T2) follow-up relative to pre-experience baseline (T1) scores. Independent samples *t*-tests were used to determine whether there were statistically significant mean differences in pre-post change scores for authoritarian attitudes across conditions (escitalopram, psilocybin).

## Results

### Descriptive statistics

In Study 1, the baseline survey was completed by 629 adult participants, of which 269 (43%) completed the 2-week follow-up survey, and 180 (29%) completed the 4-week follow-up survey. Among the 269 participants who completed the 2-week survey, 30% were female. The mean age was 32 years (range, 18–71), and 9% of the participants had not used psychedelics before the study, with most using LSD or 1P-LSD (42%), psilocybin (26%), or ayahuasca (12%) during the psychedelic experience reported in the study. In Study 2, there were 28 adult participants, of which 43% were female. The mean age was 41 years (range, 29–59), and none of the participants had used psilocybin or other psychedelics before the study. In Study 3, there were 29 adult participants who were randomized to the escitalopram condition, of which 31% were female. The mean age in the escitalopram condition was 39 years (range, 22–60), and 72% of the participants had not used psilocybin before the study. There were 30 adult participants randomized to the psilocybin condition, of which 37% were female. The mean age in the psilocybin condition was 43 years (range, 21–60), and 73% of the participants had not used psilocybin before the study.

### Inferential statistics

In Study 1, the LME revealed no significant effect of time on authoritarian attitudes (*F*_(2,262)_ = 1.56, *p* = 0.213) and *post hoc* comparisons between the time points confirmed that participants did not report changes in authoritarian attitudes at two (*M* = 1.79, standard deviation (SD) = 0.55; *p* = 0.329, 95% confidence interval (CI) [−0.02, 0.09]) or 4 (*M* = 1.79, SD = 0.60; *p* = 0.175, 95%CI [−0.02, 0.10]) weeks after the psychedelic experience, relative to baseline scores (see Table 1).

**Table 1.** Changes in authoritarian attitudes, study 1.

Interval	Mean (SD)		*t*	*p* *	*n*
T1-to-T2	1.87 (0.61)	1.79 (0.55)	1.39	0.329	269
T1-to-T3	1.87 (0.61)	1.79 (0.60)	1.71	0.175	180

*Note.* Scores are noted as means (SDs); *n* refers to number of responses. T1: Baseline. T2: 2 weeks post psychedelic experience. T3: 4 weeks post psychedelic experience. Higher mean scores: greater authoritarianism. SD: Standard deviation.

* Bonferroni adjustment applied for multiple comparisons.

In Study 2, the LME revealed no significant effect of time on authoritarian attitudes (*F*_(4,26)_ = 2.57, *p* = 0.061) and *post hoc* comparisons between the time points confirmed no changes in authoritarian attitudes at two (*M* = 2.22, SD = 0.56; *p* = 0.623, 95%CI [−0.29, 0.08]) or 4 (*M* = 2.09, SD = 0.58; *p* = 0.999, 95%CI [−0.18, 0.23]) weeks after the 1 mg control, relative to baseline scores. Similarly, no significant changes in authoritarian attitudes were observed at two (*M* = 1.92, SD = 0.62; *p* = 0.352, 95%CI [−0.09, 0.43]) or 4 (*M* = 1.96, SD = 0.62; *p* = 0.475, 95%CI [−0.09, 0.36]) weeks after the 25 mg psilocybin experience (see Table 2).

**Table 2.** Changes in authoritarian attitudes, study 2.

Interval	Mean (SD)		*t*	*p* *	*n*
T1-to-T2	2.11 (0.66)	2.22 (0.56)	1.46	0.623	26
T1-to-T3	2.11 (0.66)	2.09 (0.58)	0.28	0.999	28
T3-to-T4	2.09 (0.58)	1.92 (0.62)	1.78	0.352	25
T3-to-T5	2.09 (0.58)	1.96 (0.62)	1.61	0.475	28

*Note.* Scores are noted as means (SDs); *n* refers to the number of responses. Single-arm study. T1: Baseline. T2: 2 weeks post 1 mg psilocybin (placebo dose). T3: 4 weeks post 1 mg psilocybin. T4: 2 weeks post 25 mg psilocybin. T5: 4 weeks post 25 mg psilocybin. Higher mean scores: greater authoritarianism. SD: standard deviation.

* Bonferroni adjustment applied for multiple comparisons.

In Study 3, there were no significant within-group changes from T1-to-T2 in the escitalopram group (*t*(28) = 0.91, *p* = 0.372) or the psilocybin group (*t*(29) = 0.33, *p* = 0.742; see Table 3). There were also no significant between-group differences at either time point (T1: *t*(57) = 0.30, *p* = 0.767; T2: *t*(57) = 0.51, *p* = 0.616) or in change scores from T1-to-T2 (*t*(57) = 0.34, *p* = 0.739; see Table 4).

**Table 3.** Changes in authoritarian attitudes (within-arm), study 3.

Interval	Mean (SD)		*t*	*p*	*n*
T1-to-T2 (escitalopram)	2.18 (0.69)	2.12 (0.70)	0.91	0.372	29
T1-to-T2 (psilocybin)	2.24 (0.86)	2.21 (0.76)	0.33	0.742	30

*Note.* Scores are noted as means (SDs); *n* refers to the number of responses. T1: baseline. T2: 6 weeks after baseline. SD: standard deviation.

**Table 4.** Changes in authoritarian attitudes (between-arm), study 3.

Interval	Escitalopram	*n*	Psilocybin	*n*	*t*	*P*
T1	2.18 (0.69)	29	2.24 (0.86)	30	0.30	0.767
T2	2.12 (0.70)	29	2.21 (0.76)	30	0.51	0.616
T1-to-T2	−0.06 (0.37)	29	−0.03 (0.44)	30	0.34	0.739

*Note.* Scores are noted as means (standard deviations); *n* refers to the number of responses. T1: baseline. T2: 6 weeks after baseline.

## Discussion

In three exploratory studies with different designs and populations, we investigated the relationship between psychedelic use and authoritarian attitudes. The results showed no significant changes in authoritarian attitudes after psychedelic use, which contrasts with findings from previous research on psychedelic use and authoritarian attitudes (Lyons and Carhart-Harris, 2018), as well as common beliefs during the 1960s counterculture around the political effects of psychedelic use (Nichols, 2016). Taken together, these null findings therefore suggest that there is limited and inconsistent evidence that psychedelic use influences authoritarian attitudes in a reliable direction.

Even though we did not find evidence that psychedelic use can influence authoritarian attitudes, it is still conceivable that psychedelic use could have an effect on authoritarian attitudes under certain conditions. For example, given that the acute and post-acute effects of psychedelics appear to depend on contextual factors (e.g., mindset, social and physical environment, music; Aday et al., 2021; Carhart-Harris et al., 2018; Simonsson et al., 2023, 2025), psychedelics might act as non-specific amplifiers of the sociopolitical context(s) before, during, and after the psychedelic experience (Pace and Devenot, 2021). It is therefore possible that exposure to particular political content in close time proximity to the psychedelic experience could influence authoritarian attitudes. Future research should investigate such possibilities.

### Limitations

While the inclusion of studies with different designs and populations increases the reliability of the (null) findings, several limitations should be considered when interpreting the overall evidence across the three studies (see Carhart-Harris et al., 2021; Haijen et al., 2018; Lyons et al., 2024 for overall limitations in these studies). First, in all three studies, the mean score on authoritarian attitudes at baseline was lower than in the study that found a decrease in authoritarian attitudes following psychedelic use (Lyons and Carhart-Harris, 2018). The relatively low baseline scores of authoritarian attitudes mean there might have been a floor effect (i.e., limited potential for scores to decrease further), which may explain the discrepancy in findings. Second, no data were collected on contextual factors that could possibly modulate effects on authoritarian attitudes (e.g., the broader social and political environment, exposure to political content through social media or traditional media, political beliefs of the experience providers or facilitators). Such data may have added nuance to the results. Third, we assessed items related to authoritarian attitudes, but it is possible that psychedelics, under certain conditions, could influence other facets of political psychology, including attitudes and feelings toward the political outgroup or environmental attitudes (Gandy et al., 2020; Kettner et al., 2019; Roseman et al., 2021; Roseman and Karkabi, 2021). It may also be that authoritarian attitudes (or the questionnaire that was used in this study to measure them) are not the most sensitive to psychedelic-induced belief change. Future research should recruit larger and more diverse samples, collect additional context-related data, and also investigate political outcomes other than authoritarian attitudes.
